# Risk factors of hypocalcemia after total thyroidectomy. A high volume center experience

**DOI:** 10.3389/fendo.2025.1538993

**Published:** 2025-05-13

**Authors:** Ömer Faruk İnanç, Kenan Çetin, Yasin Tosun, Hasan Fehmi Küçük

**Affiliations:** ^1^ General Surgery Department, Anadolu Medical Center In Affiliation With Johns Hopkins Medicine, Istanbul, Türkiye; ^2^ General Surgery Department, Faculty of Medicine, Çanakkale Onsekiz Mart University, Çanakkale, Türkiye; ^3^ General Surgery Department, Kartal Dr. Lutfi Kırdar City Hospital, Istanbul, Türkiye

**Keywords:** hypocalcemia, thyroidectomy, risk factors, hypoparathryroidism, endocrine surgery

## Abstract

**Introduction:**

Thyroidectomy is one of the most frequently performed surgical procedures worldwide. The most common complication of total thyroidectomy (TT) in the early postoperative period is hypocalcemia. This study aims to determine the risk factors for postoperative hypocalcemia after TT and to reveal their clinical value. As a secondary outcome, we assessed the effects of iatrogenic parathyroidectomy, surgical experience, and parathyroid transplantations on prolonged than one month hypocalcemia and intravenous calcium infusion requirement after TT.

**Methods:**

We designed our study as a retrospective cohort study. Two hundred sixty-three patients that underwent total thyroidectomy in a single tertiary endocrine surgery unit were included. Patients are followed up for 6 months. The study performed between April 2014 and March 2015. Patients were divided into two groups according to the presence or absence of hypocalcemia after surgery. All patients who performed total thyroidectomy without lymph node dissection in a single session were initially included in the study cohorts. Thereafter, patients with preoperatively confirmed hyperparathyroidism, hypoparathyroidism/hypocalcemia, had a history of thyroid operation, and postoperatively did not undergo regular follow-up (min. 12 months after surgery) were excluded from the latter analysis.

**Results:**

In the multivariate analysis we conducted in our study, we found that female gender, preoperative hyperthyroidism, intraoperative parathyroid autotransplantation, and surgical experience were independent risk factors. Some of them are predictable parameters such as surgeon experience and preoperative hyperthyroidism.

**Conclusions:**

We consider that specific theoretical and practical studies on thyroid surgery will reduce postoperative hypocalcemia.

## 1 Introduction

Thyroidectomy is one of the most frequently performed surgical procedures worldwide (1). In the last few decades, total thyroidectomy (TT) has replaced subtotal thyroidectomy, which has a higher probability of recurrence for both malignant and benign diseases. The most common complication of TT in the early postoperative period is hypocalcemia (1.6-50%) (2). Postoperative hypocalcemia is called temporary if it lasts less than 6 to 12 months after the operation, and permanent if it lasts longer than 12 months. While temporary hypocalcemia increases the cost by prolonging the hospitalization period, permanent hypocalcemia creates permanent morbidity in the patient with the need for calcium replacement therapy. Therefore, there is interest in investigating risk factors for the development of postoperative hypocalcemia after TT.

This study aims to determine the risk factors for postoperative hypocalcemia after TT and to reveal their clinical value.

## 2 Materials and methods

The study was designed as a retrospective cohort study. Two hundred sixty-three patients that underwent total thyroidectomy in a single tertiary endocrine surgery unit between April 2014 and March 2015 were included. The study protocol was approved by the institutional review board accordingly. All patients who performed total thyroidectomy without lymph node dissection in a single session were initially included in the study cohorts. Thereafter, patients with preoperatively confirmed hyperparathyroidism, hypoparathyroidism/hypocalcemia, had a history of thyroid operation, and postoperatively did not undergo regular follow-up (min. 12 months after surgery) were excluded from the latter analysis.

Patients were divided into two groups according to the presence or absence of hypocalcemia after surgery:

1. Group hypocalcemia including patients with low blood calcium levels(<8.0mg/dL) ± clinical signs (postoperative numbness in the perioral area or hands, Chvostek Sign, Trousseau sign, or the presence of tetany).
2. Group normocalcemic including patients without low blood calcium levels and any sign associated with hypocalcemia.

All blood samples were taken from a peripheral vein postoperative the first morning at 07.00-07.30 for the visit. Our laboratory’s reference ranges of Ca and PTH in the blood are 8.4-10.4 mg/dL and 13.00–65.00 pg/mL, respectively. Despite 12 months having passed after TT, patients requiring calcium supplementation with low Ca (<8.0 mg/dL), and PTH (<13 pg/mL) levels were considered permanent hypocalcemia. The experience of the physician was based on the mean number of thyroid surgeries performed annually by general surgeons who worked at our center (n: 5) during the last 3 years before the study period (low experienced (n:3): <50 operations/year; high experienced (n:2): ≥50 operations/year). Hyperthyroidism was diagnosed as per the result of thyroid function tests (T3, T4, and thyroid-stimulating hormone) conducted before surgery. Incidental parathyroidectomy was defined as the presence of parathyroid glands in the thyroidectomy specimen pathological reports.

We determined the risk factors for postoperative hypocalcemia after TT as primary outcomes. As a secondary outcome, we assessed the effects of iatrogenic parathyroidectomy, surgical experience, and parathyroid transplantations on prolonged than 1-month hypocalcemia and IV Ca infusion requirement after TT. Suppose there is a symptom of hypocalcemia and Ca <8.0mg/dl; 2500mg calcium carbonate+9.68mg cholecalciferol ± 0.25 microgram synthetic calcitriol twice a day is started. Patients who develop postoperative hypocalcemia in our clinic are followed up once a month for the first 6 months. In hypocalcemia lasting longer than 6 months, follow-up is done every 3 months.

### 2.1 Statistical analysis

Data were analyzed with Statistical Package for Social Sciences software (SPSS ver. 24.0, IBM Co., Armonk, NY, USA). Pearson’s chi-square test and Fisher’s exact test were used to compare qualitative data. Normality for the distribution of quantitative variables was analyzed with the Kolmogorov-Smirnov test. The student *t-test* was used to compare the normally distributed data. Multivariate logistic regression analysis was performed to assess variables that may be associated with hypocalcemia. Based on the result of the analysis, p<0.05 was considered to be statistically significant.

## 3 Results

A total of 263 patients with total thyroidectomy were included in the retrospective cohort, of which 109 (41.4%) were performed by low-experienced and 154 (58.6%) were performed by high-experienced surgeons in one year. The demographic characteristics and preoperative findings of the patients are given in detail in **Table 1**.

**Table 1:** Preoperative and postoperative data of the retrospective cohort.

Total number, n	263
Female, n (%)	207 (78,7)
Male, n (%)	56 (21,3)
Mean Age ± sd (range)	50,2 ± 12,1 (17-77)
Female	49,8 ± 12,4 (17-77)
Male	51,6 ± 10,9 (30-74)
Preoperative Thyroid Hormone Status	
Euthyroid	171 (65)
Hypothyroid	13 (4,9)
Hyperthyroid	39 (14,8)
Subclinical Hyperthyroid	40 (15,2)
Pathology Report	
Benign	173
Malign	90
WDT-UMP*	3
Microcancer	49
Macrocancer	38
Iatrogenic Parathyroidectomy	61 (23,2)
Unilateral	54 (20,5)
Right	24 (9,1)
Left	30 (11,4)
Bilateral	7 (2,7)
Intraoperative Parathyroid Auto-transplantation	19 (7,2)
Surgeon Experience	
Low - Experienced	109 (41,4)
High - Experienced	154 (58,6)
Postoperative Hypocalcemia	61 (23,2)
Biochemical, Without Symptoms	17 (6,5)
Biochemical, With Symptoms	44 (16,8)
Postoperative IV Calcium infusion Need	47 (17,9)
Inpatient Service (IS)	28 (10,6)
Emergency Room (ER)	15 (5,7)
IS + ER	4 (1,5)
Type of Hypocalcemia	
Temporary	
< 1 Month	36 (13,7)
1–6 Months	22 (8,4)
6–12 Months	1 (0,4)
Permanent (>12 ay)	2 (0,8)
Hoarseness	8 (3)
Temporary	5 (1.9)
Permanent	3 (1.1)

* Well-Differentiated Tumor with Uncertain Malignant Potential.

Iatrogenic parathyroidectomy was seen in 61 (23.2%) patients. Intraoperative parathyroid autotransplantation was performed in 19 (7.2%) patients. Postoperative hypocalcemia was detected in 61 (23.2%) patients. While it was detected only biochemically in 17 (6.5%) patients, symptomatic hypocalcemia occurred in addition to biochemical hypocalcemia in 44 (16.8%) patients. Intravenous calcium replacement was performed in 47 (17.9%) of these patients; 28 (10.6%) in the emergency room, 15 (5.7%) in the inpatient service and 4 (1.5%) in the inpatient + emergency room. Hypocalcemia lasted less than 1 month in 36 (13.7%) patients, hypocalcemia lasted between 1–6 months in 22 (8.4%) patients, and hypocalcemia lasted between 6–12 months in 1 (0.4%) patient. Permanent hypocalcemia lasting longer than 12 months was detected in 2 (0.8%) patients. Postoperative hoarseness developed in 8 (3%) patients. Of these, 5 (1.9%) were found to be temporary hoarseness and 3 (1.1%) as permanent hoarseness (**Table 1**).

In the univariate analysis, female gender, preoperative hyperthyroidism, iatrogenic parathyroidectomy, intraoperative parathyroid auto-transplantation, and surgical experience were associated with postoperative hypocalcemia significantly (p<0.05) (**Table 2**).

**Table 2:** The univariate analysis of the factors associated with hypocalcemia after thyroidectomy.

Variables	Postop Hypocalcemia		P value
	No, n:202	Yes, n:61	
Age, mean± sd.	50.8 ± 11.9	47.9± 12.7	.1
Female gender	151 (72.9)	56 (27.1)	.004
Hyperthyroidism, n (%)	25 (64.1)	14 (35.9)	.04
Malignancy, n (%)	69 (76.7)	21 (23.3)	.9
Iatrogenic Parathyroidectomy, n (%)			.002
No	164 (81.2)	38 (18.8)	
Yes- unilateral/bilateral	38 (62.3) – 35/3	23 (37.7) – 19/4	
Parathyroid Autotransplantation, n (%)			.04
No	191 (78.3)	53 (21.7)	
Yes	11 (57.9)	8 (42.1)	
Surgical Experience, n (%)			<.001
High - Experienced	131 (85.1)	23 (14.9)	
Low - Experienced	71(65.1)	38 (24.9)	

The multivariate analysis revealed that female gender, preoperative hyperthyroidism, intraoperative parathyroid auto-transplantation, and surgical low experience significantly increased the probability of hypocalcemia after total thyroidectomy around 4.2 [OR (95%CI):4.17(1.49, 11.66), p:0.007], 2.5 [OR (95%CI):2.48(1.11, 5.51), p:0.026], 3.2 [OR (95%CI):3.22(1.16, 8.99), p:0.025], 3 [OR (95%CI):2.96(1.57, 5.59), p:0.001] times more, respectively - as independent risk factors (**Table 3**).

**Table 3:** The multivariate analysis of the factors associated with hypocalcemia after thyroidectomy.

Parameters	Multivariate analysis		
	Odds Ratio	CI %95	P-value
Age	0.985	0.960-1.011	0.25
Sex (Fem)	4.166	1.488-11.664	0.007
Hyperthyroidism	2.475	1.113-5.507	0.026
Iatrogenic parathyroidectomy	1.955	0.989-3.865	0.054
Parathyroid autotransplantation	3.220	1.155-8.980	0.025
Low-Surgical experience	2.962	1.570-5.590	0.001

## 4 Discussion

Thyroidectomies have become the most frequently performed surgical procedures for benign and malignant thyroid diseases. With the increasing frequency of its application, surgeons began to face the complications of thyroidectomies more frequently. The most common complication after thyroid surgery is hypocalcemia. Therefore, many studies have been conducted and presented to the literature to determine the risk factors of this complication. Although many risk factors have been identified, this issue has still not been fully clarified due to conflicting results of studies. We think that the most important reason for this is the differences in the procedures applied. In our study, we examined bilateral total thyroidectomies and endeavored to reveal independent risk factors for postoperative hypocalcemia. Due to the different criteria used to define it, there is no true incidence of hypocalcemia after thyroidectomy (3). These rates have been reported to increase up to 68% for transient hypocalcemia and up to 14.5% for permanent hypocalcemia (4, 5). Patients who do not have low calcium levels may show hypocalcemia symptoms or may not show hypocalcemia symptoms in spite of low calcium levels. Thus, in our study, we defined the hypocalcemia group (Group 1) as patients with calcium levels lower than 8.0 mg/dl, with or without symptoms in order to have an objective approach.

Factors that increase the risk of developing hypocalcemia after total thyroidectomy are known as age, female gender, pathology and duration of underlying thyroid disease, parathyroid gland tissue damage, long-term vitamin D deficiency history, iatrogenic excision or devascularization of parathyroid gland(s) (6–8). We can group them under headings such as disease-related, patient-related (comorbidities) and factors related to the management of the surgical process (9).

The age factor has been examined in many studies and contradictory results have been reported. Some studies have reported advanced age while others have reported young age as risk factors (10). Qin et al., in their meta-analysis which included 50 studies, defined age as a risk factor for transient hypocalcemia in 13 of the included studies. They found in their analysis that young age was associated with an increased rate of hypocalcemia in patients who underwent TT. They emphasized that central lymph node metastases were more common in young patients with thyroid cancer and that more dissection was needed in these patients. Puzziello et al., in their study of 2,631 patients, specified that there was no difference between the genders in terms of the development of permanent hypocalcemia after transient thyroidectomy, and that transient hypocalcemia was more common in female gender (11).

We could not find a significant age difference between the groups with and without hypocalcemia in our study. Many studies have shown that female gender is a risk factor for postoperative transient hypocalcemia development, and the effects of hormonal changes on vitamin D, PTH, and calcium absorption have been emphasized (12, 13). In our study, female gender was observed to be associated with postoperative hypocalcemia in the univariant analysis and was one of the independent risk factors identified in multivariate analysis.

It is known that patients who underwent total thyroidectomy with the indication of Graves’ disease needed more calcium replacement than patients who underwent surgery with other indications (14). In addition, it was demonstrated that as the weight of the thyroid gland increased, the risk of developing postoperative hypocalcemia increased (15). However, there are not enough studies in the literature on the effect of preoperative hyperthyroidism on the development of postoperative hypocalcemia. With this aspect, it is important that preoperative hyperthyroidism was shown as an independent risk factor in the multivariate analysis in our study.

Iatrogenic parathyroidectomy is a very common complication after thyroid surgery. Although this seems to be directly related to the surgeon’s experience, it is reported as a bad surprise in the pathological examinations of thyroidectomy specimens of even the most experienced surgeons due to local inflammation, malignant invasion, and intrathyroidal ectopic location (16). In our study, iatrogenic parathyroidectomy increased the incidence of transient hypocalcemia. However, as the number of patients with permanent hypocalcemia was quite small (n:2, 0.8%), its relationship could not be investigated. Also, the effect of iatrogenic excision of one parathyroid gland from each side on postoperative hypocalcemia compared to one unilateral parathyroid gland removal could not be examined due to the small number of patients (n:7; 4 patients in the postoperative hypocalcemia group, 3 patients in the normocalcemia group). Intraoperative parathyroid autotransplantation and the number of autotransplanted glands have also been reported as risk factors for hypocalcemia after thyroidectomy (17, 18). On the other hand, in a study conducted with 313 cases, one parathyroid gland was excised in only three patients after total thyroidectomy, and all three were autotransplanted. However, transient hypocalcemia developed in 5.4% of the patients. Persistent hypocalcemia was not reported (19). Autotransplantation into the sternocleidomastoid muscle was performed in 19/263 (7.2%) of the patients in our cohort study. Hypocalcemia developed in 8 (42.1%) of the patients who underwent autotransplantation. However, hypocalcemia was short-lived in all of them (5 < 1 month, 3 1–3 months). For this reason, we perform autotransplantation in selected cases (in the case of glands that were accidentally removed and noticed during surgery or devascularized during dissection), not routinely. In our study, we determined parathyroid autotransplantation as an independent risk factor for postoperative transient hypocalcemia. On the other hand, autotransplantation was not performed in two of our patients who developed permanent hypocalcemia. Additionally, parathyroid tissue was not reported in the thyroidectomy specimen of these two female patients, but their thyroidectomies were performed by low-volume surgeons.

In our study, we found that the experience of the surgeon was significantly important in postoperative hypocalcemia development. It is generally accepted that surgical experience affects the postoperative permanent hypocalcemia development. However, its relationship with postoperative transient hypocalcemia has not been fully clarified. In the multivariate analysis of a study conducted by Burge et al., surgical specialty and stage of thyroid carcinoma were determined as independent risk factors for the development of permanent hypoparathyroidism (20). In the same study, it was reported that 29% of the patients operated by otolaryngologists developed permanent hypoparathyroidism, and 5% of the patients operated by general surgeons developed permanent hypoparathyroidism. In a total thyroidectomy study with 111 patients, surgeons were asked to score the viability of the parathyroid glands they saw. They named this scoring, between 0–3 according to viability, as the “Index of parathyroid viability score (IPVS)”. This scoring has been shown to be associated with postoperative hypocalcemia development (21). This information explains that surgical experience is important in terms of recognizing and protecting parathyroid glands. In a study conducted in a high-volume center for thyroid surgery, thyroidectomies performed by an experienced surgeon, a young specialist and an assistant were compared. It was shown that postoperative hypocalcemia development in less than 90 days and in the long term was not significantly different between these three groups, which is not compatible with our study (22).

## 5 Conclusion

Postoperative hypocalcemia is still a common complication after total thyroidectomy. In the multivariate analysis we conducted in our study, we found that female gender, preoperative hyperthyroidism, intraoperative parathyroid autotransplantation, and surgical experience were independent risk factors. Some of them are predictable parameters such as surgeon experience and preoperative hyperthyroidism. We consider that specific theoretical and practical studies on thyroid surgery will reduce postoperative hypocalcemia.

## Data Availability

The original contributions presented in the study are included in the article/supplementary material. Further inquiries can be directed to the corresponding author.
